# The lymphatic vasculature in lung function and respiratory disease

**DOI:** 10.3389/fmed.2023.1118583

**Published:** 2023-03-14

**Authors:** Anjali Trivedi, Hasina Outtz Reed

**Affiliations:** ^1^Weill Cornell Medical Center, New York, NY, United States; ^2^Graduate School of Medical Sciences, Weill Cornell Medicine, New York, NY, United States

**Keywords:** lung lymphatics, LECs, lymphatic dysfunction, inflammation, lung disease

## Abstract

The lymphatic vasculature maintains tissue homeostasis *via* fluid drainage in the form of lymph and immune surveillance due to migration of leukocytes through the lymphatics to the draining lymph nodes. Lymphatic endothelial cells (LECs) form the lymphatic vessels and lymph node sinuses and are key players in shaping immune responses and tolerance. In the healthy lung, the vast majority of lymphatic vessels are found along the bronchovascular structures, in the interlobular septa, and in the subpleural space. Previous studies in both mice and humans have shown that the lymphatics are necessary for lung function from the neonatal period through adulthood. Furthermore, changes in the lymphatic vasculature are observed in nearly all respiratory diseases in which they have been analyzed. Recent work has pointed to a causative role for lymphatic dysfunction in the initiation and progression of lung disease, indicating that these vessels may be active players in pathologic processes in the lung. However, the mechanisms by which defects in lung lymphatic function are pathogenic are understudied, leaving many unanswered questions. A more comprehensive understanding of the mechanistic role of morphological, functional, and molecular changes in the lung lymphatic endothelium in respiratory diseases is a promising area of research that is likely to lead to novel therapeutic targets. In this review, we will discuss our current knowledge of the structure and function of the lung lymphatics and the role of these vessels in lung homeostasis and respiratory disease.

## Introduction

Historically, the lymphatic vascular system has been understudied compared to the blood vascular system, in part because of difficulty identifying these vessels and unique anatomical differences that make their characterization challenging. However, there have been significant advances in lymphatic research over the past two decades, and the importance of lymphatic function has been increasingly uncovered (1–3). In the lung, lymphatic vessels play critical roles spanning from the first breath after birth to roles in diseases such as asthma, tuberculosis, and COPD (4–9). While the importance of the lung lymphatics is starting to be understood, the precise mechanisms by which these vessels affect lung homeostasis and disease pathogenesis are not entirely clear. However, given that the lung is both constantly exposed to outside pathogens and toxins and also exquisitely sensitive to fluid accumulation, lymphatic function is predicted to play a key mechanistic role in lung function.

## Lung lymphatic structure and anatomy

**Figure 1:**
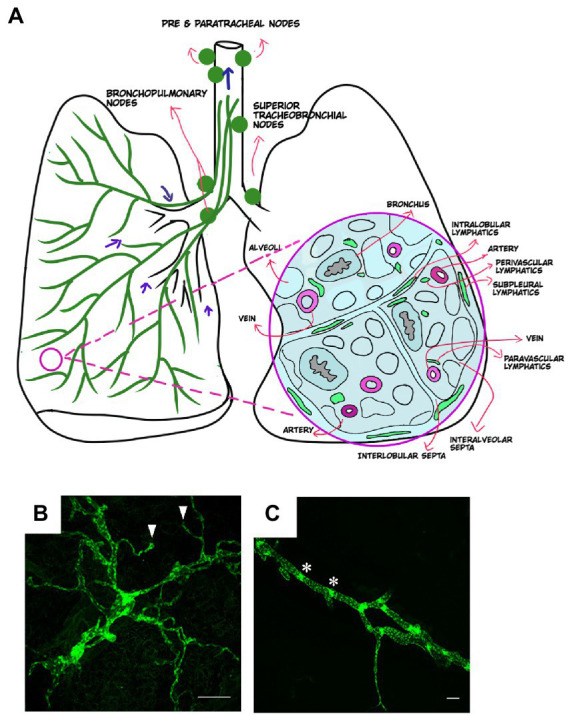
Anatomical distribution of lymphatics in healthy lung. **(A)** Distribution of lymphatics in the lung (green, left) with arrows indicating the direction of lymph flow. Cross section of the lung (right) and the distribution of lymphatics in relation to other lung structures. Images created using MediBang Paint Pro. **(B,C)** Visualization of lung lymphatics using whole mount immunohistochemistry of lung tissue from a *Prox1-EGFP* lymphatic reporter mouse, in which all LECs are labeled by GFP expression (green). Initial lymphatics are shown in **B**, with blunt ends of these vessels indicated by arrowheads. A larger collecting lymphatic is shown in **C**. Values indicated with asterisks. Scalebars = 50 um.

The lymphatic system has been described since the 16^th^ century, and landmark studies by Dr. Florence Sabin in the 1900s provided critical information about the origins and anatomy of the lymphatic vasculature during development. However, it has only been in the past 20 years that the molecular basis for lymphatic development has been uncovered. In mammals, the lymphatic system develops embryonically and is driven by specification of endothelial cells to a lymphatic identity *via* the transcription factor PROX1, which is required to bias and maintain a population of progenitor cells to the lymphatic program (10–14). Embryonic lymphangiogenesis is driven by vascular endothelial growth factor C (VEGFC) signaling through its receptor VEGFR3 on LECs (15). Lymphatic vessels form an extensive network throughout the skin and in most internal organs where they function to promote interstitial fluid drainage, trafficking of antigen presenting cells into regional lymph nodes, and absorption of lipids and small molecules (16, 17). In the lung and elsewhere, the lymphatic vascular network consists of small, thin-walled, and blind-ended initial lymphatics that drain into larger collecting lymphatic vessels (18, 19). Discontinuous button-like junctions between LECs in the initial lymphatics facilitate uptake of interstitial fluid and macromolecules (18). The collecting lymphatics have tighter and continuous zipper-like junctions and in most organs are covered with specialized muscle cells that promote lymph flow by providing contractile activity, though notably, the lymphatics in the lung lack this smooth muscle cell coverage (18, 19). Initial lymphatics in the lung parenchyma drain into collecting lymphatic vessels that are present in the bronchovascular bundles and interlobular septa (Figure 1). These collecting lymphatics drain to thoracic lymph nodes, and eventually into the thoracic duct, where lymph is returned to the blood circulation.

## Lung lymphatic development

Much of our recent understanding of the development and origins of the lymphatics are the result of studies using mouse and zebrafish embryos. In addition, the identification of mutations in several genes that are essential for lymphatic development in patients with lymphatic disorders has also aided in our knowledge of the molecular basis for lymphatic development (3). The lymphatic vasculature, including the lung lymphatics, arise from specification of LEC progenitors expressing the master transcriptional factor PROX1 in the cardinal veins to form lymph sacs (14, 20–22). Lymphatic identity in these LECs is maintained by a feedback loop involving both PROX1 and VEGFR3 (23). Migration of LECs from the lymph sacs into the developing lobes of the lungs begins at embryonic day 11.5 in mice, with more extensive patterning and lymphatic vessels with lumens detected in proximal and distal bronchovascular bundles at E14.5 (24). By E18.5, the lung lymphatic vasculature is a well-defined and continuous network that is associated with both bronchovascular bundles and veins (24).

## Identification and imaging of the lung lymphatics

Study of the lymphatic system in general, and the lung lymphatics in particular, has historically been quite challenging due to the lack of reliable imaging techniques and molecular markers as well as their thin walls, small size, variable anatomy, and complex interconnections (25). Even directly after surgical exposure, lymphatic vessels are not easily visible, as lymph is normally transparent. Studies using casting techniques in animal models provided critical early insights into the anatomy of the lung lymphatic vasculature. Using corrosion casting (26–28), lymphatics within the lung were visualized and found to consist of blind-ended initial lymphatics that empty into tubular conduit lymphatics around blood vessels and airways (29, 30). A series of ‘pre-lymphatics’ were also seen and consisted simply of tissue planes that connected with lymphatic channels on the pleural surface.

Since these studies, visualization of the lung lymphatics in animal models is now greatly aided by identification of lymphatic-specific markers and genetically modified mice that allow for microscopic analysis of these vessels. For example, lymphatic reporter mice in which LECs are labeled by expression of fluorescent proteins driven by the lymphatic-specific promoter gene PROX1 provide a key tool for identification of lung LECs (31, 32). Immunohistochemical techniques can also be used for identification of lung lymphatic vessels in human and mouse tissue using antibodies targeting lymphatic markers such as VEGFR3, PROX1, and Podoplanin. However, care must be taken in the use of these markers given organ-specific differences in their specificity for the lymphatic endothelium (33–35). For example, while relatively specific for the lymphatics in the lung, VEGFR3 is not a lymphatic-specific marker in other organs, particularly in the liver, where VEGFR3 is highly expressed in sinusoidal endothelial cells (36). Similarly, while Podoplanin can be used to detect lymphatics in the skin in mice, it lacks specificity for lymphatics in the lung, where it also expressed at high levels by type I alveolar epithelial cells (37). Expression of lymphatic markers may also change in settings of lung injury, further complicating the identification of lung lymphatic vessels (38, 39). Despite this, reliable markers for the lung lymphatics in both mice and humans have been identified, particularly when used in combination with each other (Table 1). Notably, novel radiographic techniques using inhaled lipid nanoparticles to map the draining patterns of the lung lymphatics have been developed in animal models (44, 45) and may be adapted for use in patients in the future.

**Table 1:** Lung lymphatic markers in humans and mice.

Markers	Species		Remarks
	Human	Mouse	
VEGFR3	Not specific for lung lymphatics, also expressed by blood endothelial cells	Relatively specific lung lymphatic marker, less specific in other tissues	Should be used cautiously in lung injury models where there is increased VEGFR3 expression in pulmonary capillaries
			Can stain epithelial cells and macrophages (40)
			Recommended to perform co-staining with other markers when used for human lung staining
PROX1	Can differentiate LECs from BECs	Can differentiate LECs from BECs	Detailed analysis of the tissue can be difficult given nuclear localization of this marker
LYVE-1	Not a specific marker for lung lymphatics, but can be useful in other tissues	Not a specific marker for lung lymphatics, but can be useful in other tissues	Can be combined with PROX1 for double staining to identify lung lymphatics
Podoplanin	Best marker for human lung lymphatics, especially the D2-40 epitope	Not a specific marker of pulmonary lymphatics	Also stains podocytes and epithelial cells in mice (41)
CCL21	Can be used for staining of lymphatics in the lung	Can be used for staining of lymphatics in the lung	Can also stain HEVs in mice and CD45^−^ myofibroblast-like cells in human tissue (42, 43)

LEC, lymphatic endothelial cell; VEGFR, vascular endothelial growth factor receptor; Prox1, Prospero-related homeodomain transcription factor; LYVE, lymphatic hyaluronan receptor, CCL, Chemokine (C-C motif) ligand, HEVs, High endothelial venules.

Clinically, lymphangiography has significantly advanced our ability to image the lymphatics at high resolution due to direct administration of contrast agent into cannulated lymph vessels for computed tomography (CT) of lymphatic architecture. Contrast enhanced magnetic resonance lymphangiography has also provided a great deal of new information about the anatomy of the pulmonary lymphatics, particularly in the setting of pathological lymphatic flow into the lung parenchyma (46). Newer experimental techniques combine immunohistochemistry and high-resolution micro-CT (μCT) to obtain 3D imaging and microfluidic modeling of human pulmonary lymphatics at high resolution (47).

## Lung lymphatic function

The lungs are susceptible to inflammation and injury, as they are constantly exposed to pathogens, environmental toxins, and inflammatory stimuli. Therefore, a major role of the lung lymphatics is clearing toxic substances and regulating the immune response (22, 48–50). In addition, though most fluid drainage in the lung can be accommodated *via* Starling forces to the pulmonary capillaries, lymphatic vessels clearly play a role in drainage of excess fluid and preventing edema that would compromise gas exchange (51, 52). Animal models have demonstrated that the lymphatic vessels begin draining interstitial fluid at the late gestational period, which increases compliance and changes lung mechanics to prepare for inflation at birth (9, 53).

The lung lymphatics also play a central role in coordinating the adaptive immune response by serving as a conduit for immune cell trafficking from lungs to draining lymph nodes where the response to infection and inflammation is coordinated (54, 55). Immature dendritic cells (DCs) reside in the periphery of the lung where they sample antigens found in inhaled air. These DCs extend their cellular processes between airway epithelial cells and into the airway lumen to provide continuous immune surveillance of the airway luminal surface (56–58). In addition, passive leakage of smaller antigens into the afferent lymph vessels also occurs through the tight junction barrier of LECs which acts as a molecular sieve. Lung DCs first migrate to bronchopulmonary lymph nodes and then drain to the mediastinal trunk (30). This egression of DCs from the lungs to the draining lymph nodes is tightly regulated (59), and sampling of antigens by pulmonary DCs that traffic to the lung draining lymph nodes is a key mechanism for orchestrating an appropriate immune response.

Studies using molecular tracing of DCs have proven useful for studying the kinetics of DC migration in the lung lymphatics to the draining lymph nodes (60–62). In normal conditions, a limited number of DCs reside in the lung and patrol the environment, and these cells maintain a steady state of migration *via* lymphatics to mediastinal lymph nodes every 1 to 2 days (63). Circulating pre-DCs migrate into lungs at a constant rate to replenish the resident DC pool, which turns over every 10 to 14 days (64–67). However, the turnover rate of DCs depends on anatomical location, and it has been found that the kinetics of migration of antigen presenting cells in the upper airways is much faster than in the lung parenchyma (63, 68). In one study, roughly 80% of the DCs in the upper airways were replaced by new cells within 18 h whereas only 12% of the parenchymal cells were replaced within 9 days (68). Thus, there is a balance between migration of immune cells from the lungs *via* the lymphatic vessels and replenishment of these cells from the circulation that affects lung homeostasis. Changes in the rate of lung lymphatic trafficking or in the rate of cellular influx can have profound implications on the lung immune milieu. For example, lung lymphatic dysfunction alone, in the absence of inflammatory stimuli, is sufficient to generate profound lung inflammation characterized by accumulation of lung immune cells and the formation of tertiary lymphoid organs (TLOs) (7). Conversely, in the settings of infection, lung lymphatic migration of DCs to draining lymph nodes is increased (68–73). In this way, changes in lung lymphatic trafficking coupled with the rate of immune cell recruitment to the lung can govern the inflammatory state of the lung and its response to injury.

Interactions between LECs and trafficking leukocytes also affect immune cell migration and the immune response. Chemokines provide critical cues for the directional guidance of leukocyte transmigration in lymphatics, with chemokines and adhesion molecules synthesized and secreted by LECs tightly regulating leukocyte migration along chemotactic gradients under both steady state and inflammatory conditions (59). CCL21 expression on the lymphatic endothelium and expression of its receptor CCR7 on DCs and T cells is essential for uptake and migration of immune cells *via* pulmonary lymphatic vessels (74). Immobilized CCL21 on the lymphatic endothelium also plays an essential role in directing leukocyte movement within the lymphatic vessels (75–77). Antigen presentation by DCs to the lymph nodes is therefore dependent on gradients of ligands for CCR7 on the lymphatic endothelium (62, 78–80). During inflammatory conditions, other factors such as sphingosine-1-phosphate (SIP-1), ICAM-1 and VCAM-1, and prostaglandin E2 expressed by the lymphatic endothelium are also important for leukocyte trafficking in lymphatics (38, 81–84).

## The lung lymphatics in respiratory disease

**Figure 2:**
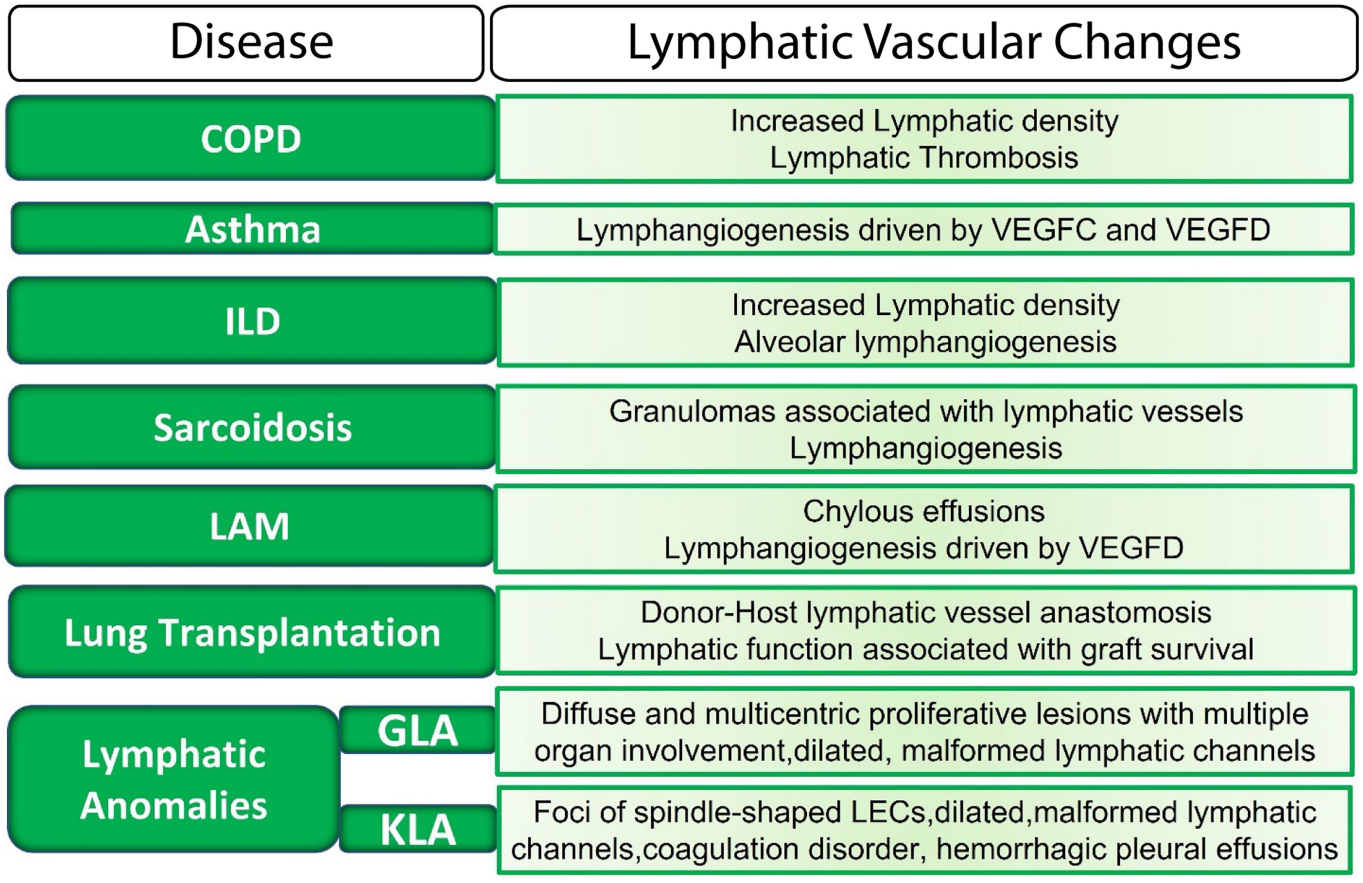
The lymphatic vasculature in lung disease. LECs, lymphatic endothelial cells; ILD, interstitial lung disease; LAM, Lymphangioleiomyomatosis; GLA, generalized lymphatic anomaly; KLA, Kaposiform lymphangiomatosis; VEGF, vascular endothelial growth factor.

Lymphatics are now considered to actively participate in physiological and pathophysiological processes in the lung, in part because of the role of these vessels in modulating the lung immune milieu. Morphological or functional defects in the lymphatic vasculature have been uncovered in many diverse pathological lung conditions (Figure 2). However, the mechanisms by which lymphatic dysfunction contributes to disease pathogenesis are not entirely clear. In some settings, lymphatic dysfunction may be secondary to lung injury and remodeling, but it is clear that in other settings, lymphatic dysfunction itself drives disease progression.

### Asthma

Asthma is an inflammatory disease characterized by mostly reversible airflow obstruction showing features such as subepithelial fibrosis, changes in the extracellular matrix, mucosal edema, and angiogenesis (85, 86). Remodeling of airway lymphatic vessels is seen in asthma (86) and in animal models has been shown to be mediated by VEGFC, VEGFD and TNF-alpha (87–89). Blockade of VEGFR3 signaling prevents tracheal lymphangiogenesis in a model of chronic airway inflammation (90). Furthermore, VEGFR3 signaling may play a role in airway inflammation by regulating the adaptive immune response (91). Interestingly, despite elevated levels of VEGFC and VEGFD, decreased lymphatic vessel density is associated with airway edema and fibrotic changes, and has been reported in fatal asthma cases (86), suggesting that anti-lymphangiogenic factors may be more prominent in severe disease. However, the exact roles that pro-lymphangiogenic and anti-lymphangiogenic factors play in acute or chronic airway inflammation are yet to be completely characterized. Further studies are required to explore the contribution of lymphatics to disease pathogenesis in asthma and how this may be targeted for therapies.

### Sarcoidosis

Sarcoidosis is a systemic granulomatous disease that is characterized by non-caseating granulomas that are found mainly in the lungs and lymph nodes (92–94). Changes in the lymphatic vasculature are associated with these pulmonary granulomas, which are distributed along the lymphatic drainage pathways in the lung. Lymphangiogenesis has been implicated in the pathogenesis of pulmonary sarcoidosis given the close association of the pulmonary sarcoid granulomas with lymphatic vessels (95–98) and the elevated levels of VEGFC and VEGFA that are seen in the serum and bronchoalveolar lavage fluid of patients with this disease. In addition, immunohistochemical staining has demonstrated that the sarcoid granulomas are a source of these lymphangiogenic factors (97, 99). Though staining for Podoplanin has revealed atypical tubular vessels around the granulomas that resemble lymphatics, they lack staining for other lymphatic markers such as VEGFR3, making their identity as lymphatic vessels a bit unclear (97).

### Lung transplantation

In case of the untreatable end stage pulmonary diseases such as COPD, pulmonary fibrosis and cystic fibrosis, lung transplantation is the only viable option available. However, long-term outcomes after lung transplantation remain poor, and acute rejection is seen in nearly 30% of recipients, which carries the additional risk of development of chronic lung allograft dysfunction (100–103). Lymphatic vessels in the lung are severed during transplant surgery and are not surgically reconnected. Despite this, reestablishment of lung lymphatic drainage has been demonstrated in both humans and animal models (104–106). Furthermore, careful analysis of the lung lymphatics post-transplant in an animal model demonstrated that functional lymphatic drainage after lung transplant is achieved *via* sprouting of donor lung lymphatics and anastomosis of these vessels with those of the host (107).

The important role of lymphatics in lung transplant can be attributed to both the function of these vessels in fluid drainage as well as immune tolerance. On the one hand, lymphatic vessels transport antigen presenting cells loaded with allogeneic antigens to the draining lymph nodes which could lead to development of immune responses leading to graft rejection. On the other hand, lymphatic vessels help clear inflammatory molecules including hyaluronan (HA), which has been shown to be associated with rejection (108, 109). Important studies using an orthotopic mouse lung transplantation model showed that simulation of lymphangiogenesis with recombinant VEGFC suppresses lung rejection and promotes clearance of HA from rejected lung grafts, improving graft survival (110). In addition, genetic deletion of the lung lymphatics in a transplant model resulted in lung inflammation and the formation of bronchus-associated lymphoid tissue (BALT) in the lungs of mice (7). Interestingly, animal models suggest that severe rejection can be detected prior to the reestablishment of lymphatic drainage (111), and furthermore, that acute lung rejection can still occur in recipients that are completely devoid of secondary lymphoid organs (112). Therefore, several lines of data offer evidence for a therapeutic role for lymphatic function in lung transplant.

### Tuberculosis

The lung lymphatics can themselves be a site of latent infection in tuberculosis (TB). Mycobacterium tuberculosis (Mtb) infection can cause lung lymphatic vasculitis that plays a role in Mtb dissemination by providing a direct route for spread from the initial site of infection in the lungs to the draining nodes (113–116). In addition, lymph nodes serve as a niche for Mtb growth and persistence (117). There is also evidence of interactions between the granulomas that form in Mtb infection and the lymphatic vasculature, as the granulomas may induce lymphangiogenesis (98), perhaps due to upregulation of a VEGFC (118, 119). In addition, extrapulmonary TB is caused by spread of the bacterium *via* the lymphatic system outside of the lung (120–122), providing a potential therapeutic target for this disease.

### COPD

Chronic obstructive pulmonary disease (COPD) is most commonly caused by cigarette smoking and is characterized by progressive respiratory symptoms and airflow limitations that are not fully reversible (123, 124). Histologic analysis of human tissue has found increased lymphatic vessel density associated with the alveolar spaces (125) as well as an increased number of lymphoid follicles in patients with advanced COPD (126). In one study, upregulated expression of CCL21 and lymphatic chemokine scavenger receptor D6 was also observed in the lung lymphatics of patients with COPD (8). Recent studies using animal models have uncovered that lymphatic dysfunction may play a role in the pathogenesis of this disease. Mice with impaired lymphatic function develop many of the histologic and pathologic hallmarks of human COPD, including hypoxia, formation of lung lymphoid follicles, and airspace enlargement due to breakdown of elastin (7). Furthermore, cigarette smoke exposure causes lymphatic dysfunction with impaired drainage, decreased leukocyte trafficking, and prothrombotic lymph resulting in lymphatic thrombosis (6). Lung lymphatic dysfunction appears prior to the development of airspace enlargement in this model, suggesting that damage to the lymphatics is an early pathogenic event in this disease. Interestingly, lung lymphatic thrombosis is also seen in human COPD, and is correlated with disease severity (6). Further studies are needed to determine the mechanisms by which cigarette smoke causes lymphatic dysfunction and how lymphatic impairment drives lung injury.

## Interstitial lung disease

Interstitial lung disease (ILD) is a debilitating disease characterized by chronic and progressive fibrosis and parenchymal remodeling. Changes in the lung lymphatics have been observed histologically in ILD, where increased alveolar lymphangiogenesis is correlated with collagen deposition and disease severity (127). In addition, though lymphatics are rarely found near alveoli in normal lungs, lymphatic vessels in the alveolar space are seen in the lungs of patients with interstitial pulmonary fibrosis, the most common form of ILD (128, 129). Severe damage of the subpleural and intralobular lymphatics has also been reported, with fragmented and disconnected vessels due to the massive fibrosis seen in this disease (130). Therefore, it is unclear whether the lymphangiogenesis seen in ILD results in functional vessels, and it is more likely that remodeling of the lung renders the lymphatic vessels unable for drain properly. Improving lymphatic function may be a novel therapeutic target in ILD, and preclinical models have indicated that stimulation of lymphatic proliferation may prevent fibrosis, suggesting a protective role for lymphatics in this disease (131).

## Lymphangioleiomyomatosis

Lymphangioleiomyomatosis (LAM) is a rare multisystem disease primarily affecting premenopausal women (132) that results from mutations in the tuberous sclerosis complex (*TSC*) genes *TSC1*, or *TSC*2 (4) in aberrant smooth muscle-like cells (LAM cells) that infiltrate the lungs, airways, and lymph nodes *via* the lymphatics (133). LAM is characterized by lymphangioleiomyomas that are comprised of the LAM cells and abnormal lymphatic channels lined by LECs (134, 135). Chylous effusions are often seen due to lymphatic dilation and dysfunction in the lungs (136). Around 70% of the patients have elevated levels of the lymphangiogenic factor VEGFD, which is a biomarker for the diagnosis of LAM (137), and is produced by the LAM cells (105). In addition, recent analysis using single cell RNA sequencing has identified transcriptional changes in LECs that suggest that crosstalk between the lymphatic endothelium and LAM cells may play an important role in the pathogenesis of this disease (138), representing a novel therapeutic target.

## Lymphatic anomalies

Complex lymphatic anomalies (CLAs) are rare disorders of embryonic lymphatic development and overlapping clinical symptoms caused by defects in the central collecting lymphatics (139–142). Generalized lymphatic anomaly (GLA) has multiorgan manifestations including in the lung, and is characterized by diffuse or multicentric proliferation of dilated lymphatic vessels (141, 143). The thoracic involvement in GLA can cause respiratory failure and is associated with poor prognosis (144). Kaposiform lymphangiomatosis (KLA) is another disease of abnormal lymphatic development and is characterized by foci of abnormal “kaposiform” spindle LECs (145, 146). Dilated and malformed lymphatic channels lined by a single layer of endothelial cells, pleural and pericardial effusions, and multiorgan involvement are common to both GLA and KLA, however patients with KLA exhibit more severe features and have coagulation disorders, hemorrhagic pericardial and pleural effusions, and some degree of fibrosis (139, 146). CLAs are generally caused by somatic mutations in genes that encode components of oncogenic growth factor signal transduction pathways, including PIK3/AKT/mTOR and RAS/MAPK (145, 147, 148). Accordingly, sirolimus, an inhibitor of mammalian target of rapamycin (mTOR), a kinase in the PI3K/AKT/mTOR pathway, appears effective at stabilizing signs/symptoms of disease in patients with GLA with mutations in this pathway (149, 150).

## Lymphatic vessels and iBALT/TLOs

Tertiary lymphoid organs (TLOs), which in the lung are also known as inducible bronchus-associated lymphoid tissue (iBALT), are accumulations of lymphoid cells and resemble LNs in their cellular content, organization, high endothelial venules, and the presence of lymphatic vessels (151–153). Lymphatic vessels and lymphangiogenesis are key features of TLOs (152, 154) and presence of iBALT are a hallmark of chronic inflammatory lung diseases (155). iBALT are not present at the time of birth, however they can develop at any time postnatally after a pulmonary insult (156). iBALT formation is seen in association with diverse lung diseases including COPD, infection, rheumatoid arthritis-associated lung disease, and lung transplantation (157–162). They can be protective for lung injury in the case of infection, where iBALT result in improved viral and bacterial clearance in animal models (163–165). Conversely, iBALT can be a source of autoreactive antibodies and autoimmune lung injury in other settings, including COPD and rheumatoid arthritis (161, 166–169). In models of lung transplantation, iBALT are associated with development of antibody-mediated rejection (170, 171), however other models indicate that iBALT may be a source of regulatory T cells that promote graft tolerance (172–174). The ways in which the pathogenicity of iBALT is regulated is of great importance for studies of the role of these structures in lung disease. Interestingly, lymphatic dysfunction alone in mice is sufficient to cause iBALT formation, in the absence of any other inflammatory insult (7). Lymphatic function is also implicated in the formation of TLOs in the gut, where TLOs restrict lymphatic drainage and immune cell migration in a model of inflammatory bowel disease (175). Crosstalk between the lymphatics and immune cells may play an important role both in the formation of these structures and their function, and is subject of ongoing investigations.

## Conclusion

Rather than passive conduits, the lymphatic vasculature is increasingly recognized for its role in modulating organ function and disease pathogenesis. Indeed, changes in lymphatic morphology or function have been observed in nearly every lung disease in which they have been studied. The explosion of research indicating the critical role of these vessels in other organs is a model on which to build more thorough and mechanistic studies of the lung lymphatics. Novel tools for imaging these vessels, identification and isolation of lymphatic endothelial cells, and animal models for manipulating lung lymphatic function will greatly aid in our ability to investigate lymphatic function in lung homeostasis and disease. Unpacking the molecular mechanisms that govern lymphatic function and how these changes in setting of lung disease will undoubtedly lead to new therapeutic targets.

## Author contributions

AT drafted manuscript and generated figures and tables. HR edited manuscript and provided guidance on content. All authors contributed to the article and approved the submitted version.

## Funding

Supported by the NHLBI K01HL145365 (H.O.R.) and the Robert Wood Johnson Foundation (H.O.R.).

## Conflict of interest

The authors declare that the research was conducted in the absence of any commercial or financial relationships that could be construed as a potential conflict of interest.

## Publisher’s note

All claims expressed in this article are solely those of the authors and do not necessarily represent those of their affiliated organizations, or those of the publisher, the editors and the reviewers. Any product that may be evaluated in this article, or claim that may be made by its manufacturer, is not guaranteed or endorsed by the publisher.
